# How gender is socially constructed in policy making processes: a case study of the Adolescent and Youth Health Policy in South Africa

**DOI:** 10.1186/s12939-022-01819-w

**Published:** 2023-02-24

**Authors:** Tanya Jacobs, Asha S. George

**Affiliations:** School of Public Health, University of the Western Cape, Cape Town, South Africa

**Keywords:** Actors, Narratives, Policy process, Framing, Gender, South Africa, Adolescent and youth health policy, Intersectionality

## Abstract

**Background:**

Gender equality remains an outstanding global priority, more than 25 years after the landmark Beijing Platform for Action. The disconnect between global health policy intentions and implementation is shaped by several conceptual, pragmatic and political factors, both globally and in South Africa. Actor narratives and different framings of gender and gender equality are one part of the contested nature of gender policy processes and their implementation challenges. The main aim of this paper is to foreground the range of policy actors, describe their narratives and different framings of gender, as part exploring the social construction of gender in policy processes, using the Adolescent Youth Health Policy (AYHP) as a case study.

**Methods:**

A case study design was undertaken, with conceptual underpinnings combined from gender studies, sociology and health policy analysis. Through purposive sampling, a range of actors were selected, including AYHP authors from government and academia, members of the AYHP Advisory Panel, youth representatives from the National Department of Health Adolescent and Youth Advisory Panel, as well as adolescent and youth health and gender policy actors, in government, academia and civil society. Qualitative data was collected via in-depth, semi-structured interviews with 30 policy actors between 2019 and 2021. Thematic data analysis was used, as well as triangulation across both respondents, and the document analysis of the AYHP.

**Results:**

Despite gender power relations and more gender-transformative approaches being discussed during the policy making process, these were not reflected in the final policy. Interviews revealed an interrelated constellation of diverse and juxtaposed actor gender narratives, ranging from framing gender as equating girls and women, gender as inclusion, gender as instrumental, gender as women’s rights and empowerment and gender as power relations. Some of these narrative framings were dominant in the policy making process and were consequently included in the final policy document, unlike other narratives. The way gender is framed in policy processes is shaped by actor narratives, and these diverse and contested discursive constructions were shaped by the dynamic interactions with the South Africa context, and processes of the Adolescent Youth Health Policy. These varied actor narratives were further contextualised in terms of reflections of what is needed going forward to advance gender equality in adolescent and youth health policy and programming. This includes prioritising gender and intersectionality on the national agenda, implementing more gender-transformative programmes, as well as having the commitments and capabilities to take the work forward.

**Conclusions:**

The constellation of actors’ gender narratives reveals overlapping and contested framings of gender and what is required to advance gender equality. Understanding actor narratives in policy processes contributes to bridging the disconnect between policy commitments and reality in advancing the gender equality agenda.

## Introduction

More than 25 years after the landmark Beijing Platform for Action, advancing gender equality remains an ongoing global priority, as signalled by the impetus and focus of the UN Women Generation Equality Forum [1–5]. Over the past decades there have been some gains and improvements towards achieving substantive gender equality, however transforming policy, structures and systems that contribute to reproducing gender and intersecting inequalities remain a priority for action, both globally and in South Africa [6–10]. Moreover, COVID-19 has made more ‘visible’, the persistent entrenchment of intersectional gendered inequalities [11–14].

The disconnect between global health policy intentions and implementation is shaped by several factors: conceptual (i.e. lack of agreement of framings of gender terms); pragmatic (i.e. approaches and capacities for implementation), as well as political (i.e. shifting away from focus on gender relations of power and social justice agendas) [15–20]. Furthermore, dynamic relationships between policy contexts, actors, content and processes also shape how gender is problematized and how ‘solutions’ are represented as part of the social construction of policies [21–24]. Actor narratives and different framings of gender and gender equality are one part of the contested nature of gender policy processes and their implementation challenges [20, 25].

Mannell identified three narratives used by development actors to construct the problem of and solutions to gender inequality: gender equality as instrumental for development; gender as women’s rights and empowerment, and gender as power relations requiring transformation [26]. These diverse understandings of gender and approaches to gender equality, led to fractured relationships between policy actors and collaboration amongst practitioners. This continues to arise in terms of debates and diverse approaches to gender mainstreaming, including involving men in gender programmes in South Africa, for example. These tensions and diverse narratives act as inhibitors to building consensus to support implementation of gender policy recommendations and are part of the complex challenges related to policy implementation and other efforts to address and transform gender power relations [26, 27].

A key factor of how gender and gender inequality is constructed in health policy is dependent on how policy actors understand, interpret and represent the problem or issue [20, 21, 28, 29]. Actor narratives articulate and structure the ideas that are part of the policy context and the notion of policy framing is how they make sense of the world, as well as policy processes [30, 31]. This process of framing is also relevant to other issues, such as sexual and reproductive health, for example. Policy actors participate in social systems that are gendered and unequal and bring their ideas, perspectives and discursive constructions to policy processes and health systems. Therefore it is important to foreground that how gender and gender equality is framed or problematised, will shape its outcomes [25, 28]. Understanding actor narratives provides an opportunity for understanding the complex mix of ideational, institutional and systems factors, operating at macro, meso and micro levels, in health policy processes [32–37].

There is a gap in terms of gender analyses of the role of actors in health policy making in the South African health scholarship since the work of Klugman (2000). Scaffolding on this and our earlier research on 15 adolescent health policy documents in South Africa [29], this paper foregrounds actors and their gender narratives in the Adolescent and Youth Health Policy (AYHP). The main findings of our earlier research shows that there was minimal integration of gender and if so, it was mostly in gender-sensitive ways, at times gender-specific, but rarely gender-transformative [38]. Further, a critical discourse analysis revealed that dominant and marginalized discourses in these documents reflect how gender is conceptualized as fixed, categorical identities, versus as fluid social processes, with implications for how rights and risks are understood. The discourses substantiate an over-riding focus on adolescent girls, outside of the context of power relations, with minimal attention to boys in terms of their own health or through a gender lens, as well as little consideration of LGBTIQA+ adolescents beyond risks related to HIV [29].

To our knowledge there is a paucity of research, both globally and in South Africa, that describes and analyses actor narratives in adolescent and youth health policy processes, thorugh a gender lens. In response to this gap, the main aim of this paper is to foreground the range of policy actors, describe their narratives and different framings of gender, as part of the social construction of in policy, using the Adolescent Youth Health Policy (AYHP) as a case study.

The results are presented in three interrelated parts. Firstly, a description of the actor landscape and how gender was considered during the processes of making the AHYP. Secondly, a description of the constellation of gender narratives of authors and proximal actors and thirdly, contextualisation of these narratives and further reflections from a range of actors working in adolescent and youth health, for advancing the gender equality in current and future policy and programmes.

## Methods

### Theoretical and conceptual grounding

**Fig. 1:**
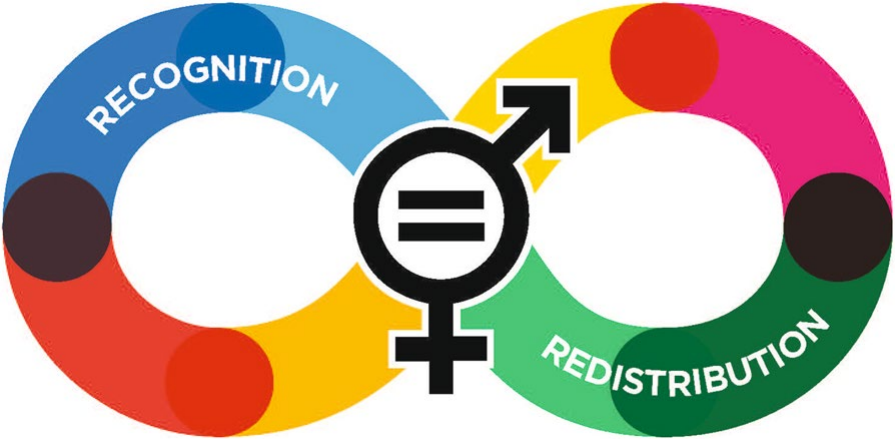
Fraser’s concepts of recognition and redistribution

**Fig. 2:**
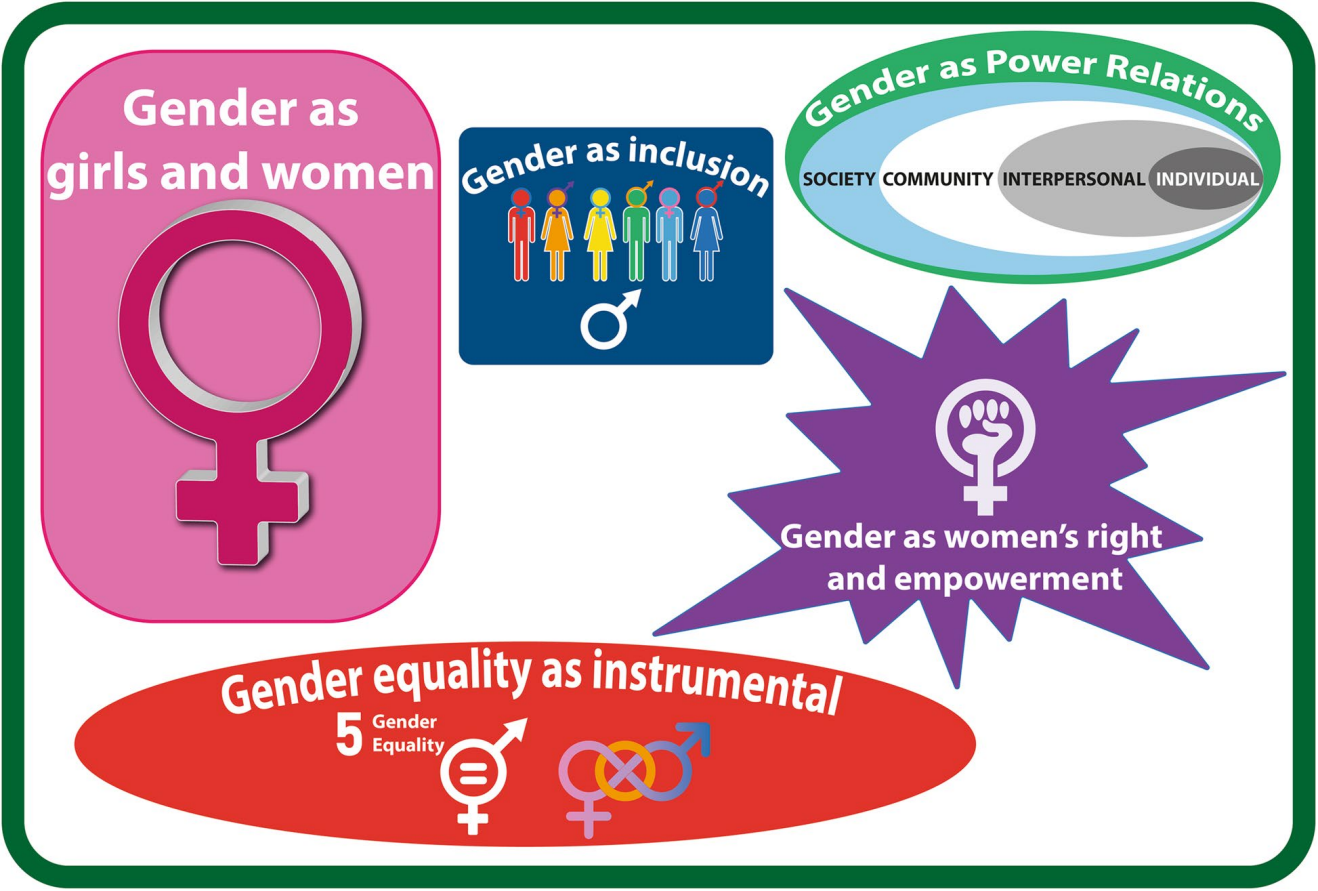
Constellation of actor gender narratives

Our conceptual grounding is guided by a bricolage approach, which combines theories and frameworks from Social Sciences (Gender Studies and Sociology) and Health Policy Analysis [39–42]. From Sociology and Gender Studies we draw on Fraser’s theoretical concepts of redistribution, recognition and representation, as starting points for ‘sense making’ of different perspectives on gender, gender inequality and gender justice [43–45]. Fraser links dimensions of recognition of identities and redistribution of resources, as gender struggles that combine the inclusionary approach and the transformative approach. (See Fig. 1) These different aspects of gender and gender equality are reflected in the actor narratives identified by Mannell (2014a), further elaborated upon in this paper. (See Fig. 2). To ensure clarity, Table 1 provides a summary of core concepts from Fraser and Mannell used in this paper and illustrated in Figs. 1 and 2.

**Table 1:** Core concepts used and illustrated in the diagrams

Concept	Explanation
Mannell [46] Gender equality as instrumental for development	Gender equality is constructed as instrumental to development objectives and economic gains
Gender equality as women’s rights and empowerment	Gender equality is constructed as equal participation of women in social, political and legal process
Gender equality as relations of power requiring personal transformation	Gender equality is constructed as being about social and power relations between men and women and embedded in the social norms and institutions
Fraser [45] Concepts of recognition and redistribution	Social justice as two separate but interrelated approaches: Recognition justice is one associated with the recognition of difference between social identities and groups (e.g., in terms of race, gender, class etc.) Redistribution justice is associated with strategies that attempt to have a more equitable distribution of resources (e.g., economic, material and political)

Sources: Mannell J. Conflicting policy narratives: Moving beyond culture in identifying barriers to gender policy in South Africa. *Crit Soc Policy*. 2014;34 (4):454–474.

Fraser N. Feminist politics in the age of recognition: A two-dimensional approach to gender justice. Studies in Social Justice. 2007;1 (1):1–5.

We also incorporate concepts and approaches from Health Policy Analysis, specifically the Policy Triangle Framework, which views policy content in conjunction with the role of actors, and how their ideas, perspectives and actions shape policy processes, as well as policy contexts [34, 36, 47]. In addition, we also draw on the notion of policy ‘frames’ and ‘framing’ by various actors and how they make sense of policy processes [30, 31, 48].

A case study design was chosen which allowed for an enquiry of the phenomenon located in the social and political context, to provide rich and thick descriptions and sense-making of the complexity and nuances that shape actor gender narratives in adolescent health policy [49, 50].

### Data collection and analysis

Qualitative data was collected via in-depth, semi-structured interviews with a range of AYHP policy authors and actors. Through purposive sampling, AYHP policy authors in government, academia and donors, members of the AYHP Advisory Panel (AP), youth representatives from the National Department of Health (NDoH) Adolescent and Youth Advisory Panel (AYAP), Commission on Gender Equality, as well as adolescent and youth health and gender policy actors, in government, academia and civil society were identified, representing a range of experiences and perspectives.

Interviews with thirty participants were conducted between September 2019 and April 2021, in an iterative manner i.e., after a first round of 15 interviews, initial analysis was conducted which guided subsequent interviews. The first round of interviews was conducted face-to-face, however in the COVID-19 context, the majority of the second round of interviews were conducted via the virtual medium which participants preferred (Zoom, Googlemeet, WhatsApp).

Thematic data analysis was guided by the literature cited earlier and interview transcripts were analysed both deductively i.e. along the lines of enquiry related to gender and actor narratives and inductively i.e. emerging issues from the data [51, 52]. In addition, interview data was triangulated both across respondents, as well as with data from the document analysis of the AYHP.

### Ethical approval

Informed consent to interview and audio record was obtained from each participant and each interview was transcribed in full. Ethical approval for the study was obtained from the University of the Western Cape (Reference number: BM 18/9/9) and each participant was assured on anonymity and confidentiality.

## Results

The results are presented in three sections: firstly, the actor landscape and how gender was considered during the process, secondly the constellation of actor gender narratives and thirdly, these actor narratives were further contextualised in terms of reflections of what is needed going forward.

### Actor landscape and how gender was considered in the process of policy making

In developing the AYHP, the actor landscape consisted of proximal actors, being the NDoH authors and academic authors as well as the UNFPA. Additional actors include youth that participated through the Mzantzi Whako research project, AYHP advisory panel members, as well as the youth members of the Advisory Panel (AYAP), established by the NDoH. In addition, more distal actors include government departments who have policies and mandates for adolescent and youth health, such as the Departments of Social Development, Basic Education, Women, Youth and Persons with Disability, the National Youth Development Agency, as well as civil society organisations and structures working in adolescent health and gender (e.g. Soul City, Lovelife, Ibis, Sexual and Reproductive Justice Coalition, and the Siyakwazi SRHR youth network.)

As part of this actor landscape, the She Conquers campaign was a significant contextual feature, developed and implemented at a similar time to the AYHP and enjoyed high level political support. This campaign was initiated to address the high HIV incidence amongst adolescent girls and young women in South Africa [53]. Some actors mentioned that the AYHP and She Conquers campaign were aligned in terms of their approach to gender due to its focus on girls and young women. However, other actors had divergent and conflicting narratives and described She Conquers as overshadowing the AYHP, creating confusion at implementation level and some also mentioned that they understood it as linked to donor-driven regional initiatives, such as DREAMS. These dimensions of the landscape, related to visibility and political priority were part of the broader policy context. In terms of gender narratives, some actors mentioned concerns that the focus was placing the burden on individual girls, to “conquer”, instead of transforming broader systems and structures that create gendered inequalities and mitigate against gender equality.

As part of the AYHP policy making process, gender was considered in a gender-sensitive, implicit way and gender power relations were not explicitly addressed. As a participant noted, a focus on direct needs superseded broader transformation goals even though participants were not averse to such broader goals:

*“I think that there is a focus on sexual and reproductive health, and of rights, I don’t think there’s a huge gendered content to it.... Where there is a gendered content, it’s through the prism of problems that girls or young women might have, and problems that boys or young men might have, rather than a conscious gender and power content to it. I think the people who were working on it, might have been very sympathetic to it, but I think the brief from the Department of Health was very much, we want something that is going to cater for youth”.* (AYHP advisory panel member_1).

Several actors mentioned that there were discussions of the impact of gender inequality on health during the policy making process, with some acknowledgement of gendered systems of power, but that these were not systematically included or operationalized in the finalisation of the policy.

*“Gender was discussed, but I don’t think that the Policy itself was very detailed on the specific gender issues that exist. But I remember that we wanted to see gender-transformative programmes and initiatives being highlighted in the policy and in practice. So, we did find that there were conversations around gender norms, harmful traditional gender practices in the country, and a whole lot more, and I think that that is reflected in the Adolescent and Youth Policy, but not as boldly as it should be. Gender equality I’m sure it’s very much highlighted there as a term, but not necessarily what it looks like for these adolescents and young people.”* (Civil society actor _20)*.*

In summary, the actor landscape was quite a complex terrain, including a range of civil society and government actors, powerful actors such as NDoH, as well as significant contextual factors such as the She Conquers campaign. Despite discussions of certain aspects of gender and certain actors wanting more gender-transformative approaches to be included, these more transformative framings were not included in the final versions of the policy document, as acknowledged by many actors. This indicates how the policy making process was shaped by the dynamic interactions between the diverse gender narratives, and how certain narratives and actors were dominant during the making of the AHYP and also further shaped by due to pragmatic reasons of the lead actors of the policy process.

### Description of the constellation of actor gender narratives

AYHP policy actors had diverse and at times conflicting, conceptual framings of gender, as part of a constellation of gender narratives. These included actor framings of gender in a range of interrelated ways: gender as girls and women, gender as inclusion, gender equality as instrumental, gender as women’s rights and empowerment and gender as power relations. These are described below and presented in Fig. 2.

#### Gender as girls and women

A dominant gender narrative expressed both explicitly and implicitly by policy authors, is that gender was framed as a focus on girls and women.

Actor narratives focussed on the key health consequences of gender inequality on girls and women. An illustrative example is, *“I think there was a big focus on the needs of adolescent girls, for the reason that they do bear the brunt of illness, in the fact that they’re the ones who get pregnant, they’re the ones who get disproportionally infected with HIV.”* (AYHP author government_2)*.* This also entailed a focus on the corresponding services needed and this was framed as, “*There is a part where the policy addresses the issue of young girls so the issues of gender, they were represented. It might not have covered all the gender related issues, but it tackled the services, like services for abuse.”* (AYHP author government_15).

#### Gender as inclusion

A linked narrative framing was gender as being inclusive of different elements. Firstly, some actors referenced sex-disaggregated data as demonstration that gender considerations were integrated in the AYHP. Woven in to this, was the actor narrative that gender is about inclusion, equal focus and participation of binary categories of boys and girls and numeric parity. This narrative of gender as quantitative parity, somewhat tied to sex-disaggregated data, was reflected by policy actors to include an inadequate consideration of adolescent boys and young men, being ‘invisible’, beyond Medical Male Circumcision services, as illustrated by the following quote*:*

*“I think the way in which this policy is written, including in the section on sexual and reproductive health, is far more on young women than on young men. I think we are now beginning to realize that you need to focus on young men as well. I think we’ve left young men behind and we only catching up on that in the HIV program. I’m a foregrounding HIV, because I think we’ve done much more thinking around these issues in HIV than anything else. When we started the She Conquers campaign, we didn’t mention young men at all. Even though if you look at the diagram that shows the route of transmission you will see men there.”* (AYHP author government_14).

Separately, more distal actors mentioned the complex interplay of diverse youth identities and multiple forms and axes of inequality as part of the notion of inclusivity as follows, “*Youth are not just a marginalized group, you need to look at the diversity of youth. There’s young boys, there are young girls, there is the different age groups, there are those with different orientations, there are those who come from different parts of the country, including the rural areas. Their perspectives are so different.” (*Government actor*_28).*

The narrative of *gender as inclusion* was articulated by policy authors to be interrelated with framings about the inclusion at times of the LGBTIQA+ community:

“*So during these youth engagements, we were having both representatives, we were having males and females. Some of them were from the LGBTIQA+ community, but both males and females were represented. So it took care of the gender sensitivity.*” (AYHP author government _6).

This actor narrative of the inclusion of LGBTIQA+ individuals was still very much described as a group to ‘add on/in’. It was linked to the recognition that discrimination of the basis of sexual orientation and gender identity and expression (SOGIE) and homophobia is very present in South Africa and is a significant barrier for gender diverse young people to access services and experience full rights as equal citizens.

The actor narratives of *gender as girls and women and gender as inclusion* (whether in terms of disaggregating by sex, parity with boys, or inclusive of diverse adolescents including LGBTIQA+ all tended to consider gender as a binary, fixed and essentialised category. They underplayed how fluid and dynamic gender relations and identities are, shaped by the social and structural systems of patriarchy, racism, sexism, social economic and other intersecting inequalities in the South African context.

#### Gender equality as instrumental

Actor narratives that focus on *gender equality as instrumental* were not explicitly mentioned, but rather inferred and referenced in broader national policies such the National Youth Policy. Despite *the gender equality as instrumental* narrative as more ‘silent’ in the AYHP, there were related actor framings of adolescent health as social and economic assets for adolescent and broader national development, as echoed in the in the following quote:

“*I think that if we are going to truly address adolescent and youth health we need increasingly to think about them as the next generation of entrepreneurial fourth industrial revolution workforce. We have the capacity to think of youth health as a springboard for the country’s enormous success. So it is not just about the fact that they don’t have HIV or they don’t have malaria. It is about having a next young generation who have the kind of capacity to fly.”* (AYHP author academic_10).

#### Gender as women’s rights and empowerment

As part of the constellation of narratives, the *gender as women’s rights and empowerment* actor narrative was relatively absent and mentioned in terms a number of challenging contextual factors. Actors mentioned that the policy context included a number of intersecting factors: lack of prioritisation and operationalisation of gender equality by government bureaucracy, weakened civil society, lack of organised youth activism, dominance of the HIV sector, as well as closing of spaces to focus on gender and intersectional systems of inequality. The following quote gives insight into this context, “W*e’ve got a good Constitution and we’ve had some progressive people in critical places…[but] there aren’t shared values among bureaucrats, and the things that push bureaucrats and the things that determine bureaucrats’ interests, aren’t necessarily anything to do with gender equality. So, it just seems to me sad that there was this kind of hope and commitment, and I think it’s slowly ebbed away, really after the Mandela era. The close relationship between civil society and people in government collapsed, and government became much more of a bureaucracy, not feeling any accountability to civil society groups, and not recognising what level of support they could get. There wasn’t a force, neither on gender issues, women’s rights generally, nor on sexual reproductive health, if you compare it to the brilliant strategic work that went on in relation to HIV.* (Civil society actor_18).

A narrative of *women’s rights and empowerment* also cannot be asserted in isolation from South Africa’s history of institutionalised racism and violence. Nested in this broader context, the epidemic of gender-based violence highlights the need to address constructions of masculinities, and transformative work with men. A quote that underscores this perspective is, *“Apartheid and the patriarchal bargain made men feel they have lost power in the democratic South Africa. They use violence to achieve that control. I think that post-apartheid a lot of men feel very betrayed and let down. They have lost a lot of authority; they have lost authority over the women and the youth. And there is a kind of a crisis of patriarchs and men are not going to stop brutalizing each other and women, while they still feel so powerless. How do we uplift people so that violence isn’t how you assert your power?”* (AYHP author academic_9).

#### Gender as power relations

Included in the constellation of actor narratives, was the *gender as power relations* narrative, being about gender as relational, requiring social transformation and disruption of system of power and inequality. This actor narrative, co-existed, but somewhat in juxtaposition and divergence with the *gender as girls and women* narrative. As mentioned earlier, this narrative was not dominant amongst AYHP authors and proximal actors and did not gain much traction in the AYHP policy making process. In addition, some actors involved in the making of the AYHP, expressed that gender as power relations was not comprehensively understood and conceptualised by the NDoH, as lead policy author, and hence this shaped the content and process of the AYHP:

“*So I don’t think gender is even understood or talked about. It is silent in terms of gender in relation to the social constructs of masculinities and femininities, about power relations. I don’t think it is on the horizon and understanding of most people in the NDoH.*” (AYHP advisory panel member_3).

Another policy actor expressed the relative absence of *gender as power relations* narrative in the final policy. In addition, intersectionality, as multiple forms of inequality which compound and exacerbate each other, was only mentioned by a small minority of actors, *“I think in a few places gender inequality is explicit, when it comes to HIV and maybe pregnancy. But it’s more in the way that these are problems, and some of them are gender driven. I don’t think there’s consciousness of intersectionality beyond that. I don’t think there’s much focus on if a person is also disabled or if they are LGBTIQ, anything of that sort. So, I don’t think it takes an approach that says, let’s look at diversity and intersectionality. I think it recognizes some of the main divisions, like rich-poor, which in South Africa, is very race based.”* (AYHP advisory panel member_1).

Further, reflections from more distal actors included the importance of addressing gender inequality, homophobia and other axes of inequality and power relations, as part of addressing health of young people in South Africa: “*One cannot talk about HIV without being aware, particularly if you are looking at the factors that put young people at risk, that gender relations and power plays a very important role. One can also not ignore the fact that the LGBTIQA+ community is also disproportionately affected, as well as learners with disability. In our responses it will become very important for us to really begin to deal with these issues of power relations, having a rights-based approach.*” (Government actor_17).

In summary, the constellation of actor narratives are diverse and contested with a lack of shared understanding of framings of gender amongst actors. The most dominant narrative was of *gender as girls and women*, with mentioned thereafter. Narratives of *gender equality as instrumental, as women’s rights and empowerment, or as gender power relations*, while articulated by some policy actors, remained largely absent from the adolescent and youth health policy making process.

### Looking back and moving forward: contextualizing the gender narratives

When asked to reflect on the status quo in order to look forward in terms of gender and adolescent and youth heath in South Africa, a broad range of actors contextualised some of the narratives described in the previous section. Three key themes emerged and include firstly, the importance of gender-transformative  and intersectional approaches, secondly, examples of how to implement these more gender transformative approaches and thirdly, the commitments and capabilities needed to take such work forward.

### Importance of gender–transformation and intersectionality on the agenda

Partly in recognition of the past challenges of advancing gender in a deeper and transformative manner, looking forward distal policy actors to the AHYP emphasised the importance of addressing compounding and intersectional inequalities in South Africa. This is captured in the following quote, “*So the political commitment and the rhetorical commitment is there, but the commitment of the resources, and the institutional capacity and the knowledge and the other mechanisms tend to be weak. South Africa is a developing country, so it has to juggle all kinds of priorities. There are huge levels of poverty in this country, huge amounts inequality, not just gender inequality, racial inequality, all kinds of inequalities that the country has to grapple with.”* (Government actor_30).

Certain tensions and challenges related to siloed ways of working and lack of intersectional approaches, for example, were mentioned by a few actors, in terms of working with gender and intersectional approaches:

“*I think is there is a long standing tension around gender in any kind of development or social program in terms of mainstreaming. So you have the gender sector, the disability sector and …and we are not going to carve out separate spaces, we are going to mainstream gender, mainstream disability, mainstream whatever else. That is not…just conceptually not a feasible plan and politically it is not a plan, if those sectors are competing for resources and attention. So it has to be a different way to imagine what the state is, what the community is, what the intervention is, that uses some set of cross cutting principles or some way to imagine the world that then does less injustice to issues of gender or disability or race.” (AYHP* advisory panel member_13).

Importantly, actors also advocated for more learning, reflection and detailed intersectional gender analyses, at micro, meso, as well as macro levels. An exemplary quote is, *“I don’t think we have ever sat down to look at where we come from and the underlying historical forces that still shape where we are going. I mean this country came from a violent patriarchal background that still holds true. There is a mantra about toxic masculinity, but some of these have become clichés. These have turned into slogans, there is no deep analysis and deep thought behind them. Our debate is shallow and we haven’t addressed some of the underlying causes of why South Africa is where it is. We are going to repeat our past because we don’t have effective ways of reflecting some of the dynamics that caused us to be where we are today.”* (Civil society actor_21).

### Examples of more gender-transformative programmatic approaches

A second and related theme that emerged in looking forward was the need for more gender-transformative, feminist and multi-component programmatic work to address the inherently patriarchal, homophobic and unequal society. In the South African context, GBV programmes were used as an example of how to include boys in gender-responsive programming, as part of moving towards transformation of gender power relations: “*So firstly we need to involve the adolescent boys and use different approaches, because they don’t want to be approached in the same manner we approach women. So you need to have programs that are relevant to addressing different age groups of males, instead of just targeting young women and girls.”* (AYHP author government_15). Actors also highlighted that gender-transformative feminist work needs to focus on masculinities, starting with young ages and across generations: “*It is bigger than just speaking to the boys, it is speaking to the fathers, speaking to the religious leaders, speaking to the cultural leaders. All of these are grooming young boys into becoming these dominant men in the society.”(*Civil society actor*_*23).

In addition, many actors mentioned Comprehensive Sexuality Education (CSE) as an important example of a structural and more gender transformative programmatic approach. This was described as follows, *“I mean there is no question that CSE is a necessary and important element of addressing gender matters. South Africa has committed to having compulsory, comprehensive sexuality education in the curriculum and it is something that we must hold our government accountable for. Now, implementation assumes a number of things. It assumes that there is a clear, age appropriate curriculum, that teachers have sufficient skill and content knowledge to be able to deliver this curriculum. It also assumes that there are available learning resources for teachers as well as for the learners, like we have for Mathematics and Geography and sufficient time to cover the necessary building blocks. Each of those things needs commitment and needs delivery to be able to achieve the ultimate goal.”* (Government actor_17).

Further, actors emphasised that an essential component of CSE is not reinforcing patriarchal systems, to be grounded in lived realities of young people and to be implemented by capacitated adults. The following quote captures this point, “*So, if I was to be the Minister, I would introduce the syllabus that educates them about sex and also teach our teachers to be comfortable about teaching and speaking about sex in schools, because a lot of teachers are not comfortable. All they teach about is what they read in the book, and when you read it in the book it is, it doesn’t come as effective as it should be when you are a student listening to someone who is educating you about it. So I believe that introduction of the new syllabus is what would really bring change to the health of youth*.” (AYAP member_29).

### Commitments and capabilities to take the work forward

Given the diverse narratives, actors also emphasised the importance of developing a shared vision, political will, high level leadership by government, and institutional capabilities to lead and co-ordinate, with commitment at all levels to mainstreaming a more feminist gender agenda that advances gender and intersectional transformation. The processes and contexts needed going forward articulated by several actors, included having collaboration and alignment within government and with civil society to encourage collective work and accountability for this more political feminist agenda:

*“We need to build broad alliances around gender, gender-based violence and sexual orientation and gender identity. So for me one has to keep much more feminist and a much more critical agenda alive.*” (Government actor_22).

Moreover, key messages from actors include that ‘working with gender’ and notions of transformation should include both top-down and bottom-up processes with ‘messy middles’, which create platforms for working with the nuances. Importantly, actors noted that these spaces need to be well-facilitated and centre representative youth structures and gender movements, which can be quite challenging in a contemporary context dominated by individual social media influencers and lack of representative youth citizenry and an organised women’s movement. Consequently, actors also called for working with complexities and intersectionality of gender and other axes of power in the South African context, by bringing together various actors in government, youth representative structures and civil society working in gender. This is important for (re-)creating of spaces for discussion, activism, building alliances and findings way of operationalising policies aimed at addressing gender and intersectional inequalities.

## Discussion

In summary, this paper describes the actor landscape during the development of the AYHP, examines the constellation of actor gender narratives involved and explores the implications of the actor landscape and narratives framings for policies and programmes moving forward. Despite the commitment to gender equality and more gender-transformative approaches being discussed during the policy making process, these framings largely did not materialize in the final policy. Upon further exploration, diverse and juxtaposed actor gender narratives co-existed in an interrelated constellation further shaped by pragmatic decision making, resulting in dominant narratives prevailing in the final policy document. These narratives were further contextualised by actors as to what is needed going forward to advance gender equality in adolescent and youth health policy and programming.

### Implications for gender in adolescent and youth health policy

The analysis makes visible the constellation of multiple actor narratives and framings of gender in the AYHP, which have implications for how gender is addressed in adolescent and youth health policy and more broadly, for gender equality and social justice in the South Africa context. The analytical insights build on the foundations of Mannell, who also highlighted the tensions between narratives and how they impact and inhibit the uptake of gender policy recommendations and collaboration between actors. From the constellation of actor narratives and framings of gender and gender equality, it is evident that actors have different starting points when talking about gender, which informs how they respond to situations. Fraser’s concepts of recognition, representation and redistribution help to understand the underlying motivations behind these diverse actor narratives and framings. Some actors understand gender to be about the recognition of identities, some about equal representation, and some about the redistribution of power and resources [43–45]. Given the complexity of transforming gender power relations and sustaining gender equality, recognising the co-existence of these narratives as potentially complementary rather than in competition with one another is critical [54]. This is a central to understanding how gender is socially constructed and surfacing the co-existence of diverse narratives is part of creating spaces for dialogue and critical engagement on what this means for health policy and systems, as contested policy narratives can undermine uptake of policy recommendations.

In deconstructing actor narratives, the dominance of the *gender as girls and women* narrative, both reflects and reproduces dominant societal narratives in South Africa, which are largely that gender is equated to biological sex, binary, further shaped by the social and structural systems of patriarchy, racism, sexism, social economic and other intersecting inequalities. Importantly, most of the identified actor narratives focusing on girls and young women did so outside of power relations i.e. as decontextualised and depoliticized. These actor narratives mirror the discourses across 15 adolescent health policies in South Africa [29], which are predominantly gender-sensitive and respond to consequences of gendered inequalities, rather than being gender-transformative. While responding to health consequences of gender inequality is an important part of the NDoH mandate, we would argue that in order to move beyond the status quo of adolescent health in South Africa, we need both gender-responsive, as well as gender-transformative policy and programmes, focussing on the broader societal context and disrupting gender power relations, as part of moving towards a gender equal and just society [55–57]. This is critical to ensure that both the interrelated ‘symptoms’ of gender inequality, such gender-based violence and HIV are addressed, as well as the underlying drivers and determinants as part of a prevention i.e. being both gender-responsive and gender-transformative.

Also, the actor narratives of gender as inclusion through consideration of essentialised identities, highlights that this can lead to a binary and competing agenda on women’s/girls’ and boys’/men’s health, without consideration and analysis of underlying gender inequalities [5, 54]. This emphasises that categorical and binary understandings of gender are now inadequate and these can lead to zero-sum arguments for competing agendas and resources, and so diluted efforts to advance gender equality [5]. Moreover, the findings highlights the tensions in addressing gender in policy and programmes and that ‘gender work’ is both technical, but also about power and hence deeply political work [12, 14, 58–61]. This talks to some of the global debates and paradigm shifts that have taken place over time from Women in Development (WID) to Gender and Development (GAD) approaches [10, 62, 63] as well as the overall challenges related to mainstreaming of gender. The findings also speak to some of the tensions for actors of how to both address the practical gender needs (i.e. be more gender-responsive), as well as address the strategic gender needs (i.e. be more gender-transformative) within programmes. Importantly, the findings also foreground critical questions for how we work with gender in adolescent and youth health, beyond binaries, heteronormativity, and notions of ‘vulnerability,’ which are often not problematised in policy and programmes.

### Actors and the policy making process

Our findings add depth and nuanced understanding of the dynamic and complex relationships between the actors, South African context, policy content and processes, in shaping both how gender is problematized or framed and how this is related to how ‘solutions’ are represented in policy processes [20, 21, 28, 64, 65].

Firstly, in terms of the dynamic policy making process, our findings foreground the role of actors, providing in-depth insights into the range of actor narratives, how these interacted during the process and which made it the final policy document. These findings, which explore the relationship between the South Africa context, characterised by historical and contemporary racism, sexism, intersecting inequalities and the actor narratives, show how actor narratives are contextualised and constructed by broader societal narratives and in turn shape policy processes, such as in other contexts for example Tanzania, Ghana and Nepal [33–35].

Secondly, the findings provide insights into the *how* of policy making, particularly how the *gender as girls and women* narratives was dominant and more gender-transformative narratives ‘evaporated’ and did not make it to the final agenda and policy document, due to lack of shared conceptual framing, pragmatic institutional processes and divergence from the *gender as power relations* narratives. Making visible the role of ideas and actor narratives contributes to explain some of challenges in addressing gender and gender equality in health policy processes. Possible additional reasons for this could be the absence of a gender transformation champion, as well as policy processes and institutional spaces that did not draw on the gender expertise in civil society and academia, as also noted by Daniels [66]. The empirical literature gives insights on the commitments and capabilities needed to take more feminist work forward. For example, as argued by Ravindran et al.*,* (2021) in a review of gender mainstreaming in UN Agencies, it is important to have a robust gender architecture, and a cohesive gender capacity-building policy system. In addition, they note the importance of learning from practice, and making visible and developing strategies to challenge embedded patriarchal organisational norms and systems.

The constellation of actor gender narratives helps to unearth individual and organizational assumptions about, and ideological commitments to, gender equality and transformation of power relations, nested in broader social systems [23, 24]. Importantly, it shines the light on the pervasiveness of patriarchy and hegemonic ideas related to gender that infuse policy processes and different types and levels of power i.e. power through institutional positions and norms, power over ideas in terms of certain ideas, being accepted and rejected and power in ideas in terms of reproduction, as also documented by Acosta, Carstensen and Friel [32, 67–69]. In focussing on actor policy narratives this paper contributes to the discussions on the ideational power of actors, lack of understanding about gender concepts and language, pragmatic limitations in policy processes and deepens the understandings of policy processes related to gender mainstreaming. This is similar to what is argued in the literature by Lombardo et al.*,* (2017) and Mazur (2017) in terms of the complexity of how gender is socially constructed in policy process and the need for more feminist analyses of policymaking processes.

Given the contested nature of gender narratives there is a need for consideration of the tensions and dynamics between dominant ideas, frames, narratives, and values which can shape priorities, competing agendas and power relations amongst actors, i.e. government, civil society, academia and young people, as also described by Gaventa [70] and Harris [71]. Further, our findings contribute to the debates on policy processes, policy coherence, as well power relations between actors [72, 73], when working towards a gender equal and just society. Reimagining a healthier future for adolescents and youth in South Africa and globally will require including adolescent and young people in their diversity as key actors, as well as investing in intersectional feminist movements that hold government accountable, building on incremental steps and lessons learned to date.

### What does this all mean in practice?

The findings raise significant implications for addressing gender (in)equality, as an important cross-cutting social and structural determinant of health for adolescents and young people and the need to both respond and address the underlying root causes [74, 75]. While there is no quick-fix solution to achieving gender equality, we however would advocate for enhancing both the technical and political competencies of health and other sectors to be able to address the nuances and complexities of gender and intersecting inequalities.

Further, key message from the findings is that CSE is an important example of a gender-transformative structural and systems level intervention, requiring collaboration between the education and health sector in government and with civil society, also centering voices and realities of adolescents and young people in their diversity [76–79]. We therefore advocate for the health sector to also focus on programmes and partnerships with other actors that address social and structural systems. This should include sectors such as education and civil society, through for example, CSE and other gender-transformative collaborations that address gender-inequality as an underlying determinant of health [80, 81].

Enhancing capabilities and building policy communities and actor alliances using governance approaches, are critical for brokering multisectoral action for collaboration, as part of bringing more feminist narratives to policy making processes [72, 82–85]. However, certain challenges remain, as civil society and women’s movements, as key actors in South Africa, have changed over time, are more fragmented and have different foci [86–88]. This raises the importance of actor management, intergenerational dialogues and talking about points of commonality as well as divergence, which are aligned to current global debates, for example in terms of meaningful participation of diverse youth.

Getting and sustaining gender on the agenda of the NDoH and other national government departments will also require recognizing the diverse actor narratives, development of shared understandings and conceptual framings different aspects and dimensions and creating spaces for building relationships between key actors. This will also entail enhancing the capacity and skills, beyond narrow framings of gender  as ‘tick box’ additions, to understanding the broader social and political context, and being able to develop and implement more gender-transformative processes and interventions, and further critical engagement with power systems [18, 29, 89]. This builds on the arguments by feminists who call for a focus on transformation of gender power relations, as well including women’s movements in policymaking [18, 63, 90]. Taking this forward is urgent, also terms of developing gender-responsive transformation, particularly in the context of COVID-19 [12, 83, 91, 92].

### Gender and intersectionality on the research agenda

The contestations in the constellation of actor gender narratives is a significant foundational theme and it is crucial to unpack what this means for both policy analysis and praxis, as part of working towards a gender-equal and just society. Therefore, there is a need for further gender and intersectional analyses and scholarship on the role of actors in health policy analysis, exploring their narratives, experiences, power relationships and potential resistance to gender equality transformation. More in-depth actor-centric analyses, including exploring their ideologies and lived gendered experiences will enrich the understanding of the complexities of policy processes and gendered systems of power.

Also, research opportunities could include doing more theoretical and empirical work on gender and power analyses of different actor groups, to learn more about policy processes and how best to make it more visible [93, 94]. Importantly, there is a need for more gender and intersectionality research to explore the different dimensions of policy contexts and power, including the role of history, politics, geographical locations and intersecting social identities and locations to generate transformative insights into structural determinants that shaped the health of adolescents [95–99]. Our research has provided a case study of poststructuralist policy analysis of the social construction of gender narratives as complex and contested and shaped by broader contexts and policy processes. This work could be the foundation for expanding the boundaries of HPA and do further research on gender and intersectionality, as features of power [95, 97, 100].

This paper has both strengths and limitations, with some of the strengths being related to researcher’s positionality, which includes more than 20 years of contextual knowledge and programmatic experience and this grounding also enabled access to a range of policy actors. However, limitations include that the analysis was retrospective with actors having to remember back to 2016/2017. The COVID-19 pandemic also created an additional demand for some actors and hence created delays in participating in the research.

## Conclusions

We took a magnifying glass to the AYHP as a case-study and the research findings provide a foundation for further insights, meta analyses and implications for gender  and gender-transformation in other health policy and programmes as well analyses of actors in policy process.

The way gender is framed in policy processes is shaped by actor narratives, and these diverse and contested discursive constructions were shaped by the dynamic interactions with the South Africa context, and processes of the AHYP. This paper contributes to the scholarship on actors’ gender narratives and critically engages with what the implications are for gender transformation and intersecting power relations in adolescent and youth health policy and programmes. Advancing the gender equality agenda, both globally and in South Africa, should include analysis and attention to how actors understand and address gender as part of socially constructed policies. The mapping of the constellation of narratives is an important foundation for further analysis and action. Gender equality is an imperative for future health and wellbeing of young people and re-conceptualizing the role actor narratives can contribute to bridging the disconnect between policy commitments and reality in advancing the gender equality agenda.

## Data Availability

Data for this paper included interview data from participants and these interviews were transcribed in full. These are available from the corresponding author on request.
